# Development and Validation of a Liquid Chromatography High-Resolution Mass Spectrometry Method for the Simultaneous Determination of Mycotoxins and Phytoestrogens in Plant-Based Fish Feed and Exposed Fish

**DOI:** 10.3390/toxins11040222

**Published:** 2019-04-13

**Authors:** Amritha Johny, Christiane Kruse Fæste, André S. Bogevik, Gerd Marit Berge, Jorge M.O. Fernandes, Lada Ivanova

**Affiliations:** 1 Toxinology Research Group, Norwegian Veterinary Institute, 0454 Oslo, Norway; christiane.faste@vetinst.no; 2 Nofima—Norwegian Institute of Food, Fisheries and Aquaculture Research, 5141 Fyllingsdalen, Norway; andre.bogevik@Nofima.no; 3 Nofima—Norwegian Institute of Food, Fisheries and Aquaculture Research, 6600 Sunndalsøra, Norway; Gerd.Berge@Nofima.no; 4 Faculty of Biosciences and Aquaculture, Nord University, 8049 Bodø, Norway; jorge.m.fernandes@nord.no; 5 Chemistry Section, Norwegian Veterinary Institute, 0454 Oslo, Norway; lada.ivanova@vetinst.no

**Keywords:** Atlantic salmon, zebrafish, liquid chromatography high-resolution mass spectrometry, mycotoxins, phytoestrogens, plant-based feed

## Abstract

New protein sources in fish feed require the assessment of the carry-over potential of contaminants and anti-nutrients from feed ingredients into the fish, and the assessment of possible health risks for consumers. Presently, plant materials including wheat and legumes make up the largest part of aquafeeds, so evaluation of the transfer capabilities of typical toxic metabolites from plant-infesting fungi and of vegetable phytoestrogens into fish products is of great importance. With the aim of facilitating surveillance of relevant mycotoxins and isoflavones, we have developed and validated a multi-analyte LC-HRMS/MS method that can be used to ensure compliance to set maximum levels in feed and fish. The method performance characteristics were determined, showing high specificity for all 25 targeted analytes, which included 19 mycotoxins and three isoflavones and their corresponding aglycons with sufficient to excellent sensitivities and uniform analytical linearity in different matrices. Depending on the availability of matching stable isotope-labelled derivates or similar-structure homologues, calibration curves were generated either by using internal standards or by matrix-matched external standards. Precision and recovery data were in the accepted range, although they varied between the different analytes. This new method was considered as fit-for-purpose and applied for the analysis of customised fish feed containing wheat gluten, soy, or pea protein concentrate as well as salmon and zebrafish fed on diets with these ingredients for a period of up to eight weeks. Only mycotoxin enniatin B, at a level near the limit of detection, and low levels of isoflavones were detected in the feed, demonstrating the effectiveness of maximum level recommendations and modern feed processing technologies in the Norwegian aquaculture industry. Consequently, carry-over into fish muscle was not observed, confirming that fillets from plant-fed salmon were safe for human consumption.

## 1. Introduction

Global fish production reached more than 171 million tonnes by 2016, of which 88% were directly used for human consumption and 12% (20 million tonnes) were used for the production of fishmeal and fish oil in aquaculture [1]. Fish and fishery products are an important source of essential nutrients in the human diet, and demand is growing in line with the increasing world population [2]. Aquaculture is the fastest-growing food industry and the intensification of the production depends on the utilisation of other resources for aquafeeds than fishmeal, for which exploitation is reaching an unsustainable level. Therefore, agricultural crops, mainly legumes, cereal grains and oilseeds, have been introduced in steadily increasing amounts into fish feeds, completely or partially replacing marine protein sources [3]. 

Plant protein sources mainly include soy, pea, lupine, alfalfa, wheat, corn, rape seeds, sunflower seeds, cotton seeds, sesame seeds, mustard oil cake, and white leadtree leaves [4]. Moreover, proteins from insects, microalgae, krill and single-cell proteins have been explored as replacements for fishmeal, but plant proteins are by far the most used ingredients in feed in aquaculture. The considerable changes in the diet composition of farmed fish include ingredients with physicochemical properties that potentially could lead to challenges regarding fish health and welfare, and product quality [5]. However, new processing technologies for plant protein extraction of undesirable components such as fertilisers, pesticides, persistent organic pollutants and heavy metals have allowed the transition from marine to agricultural sources [6]. The growth performance of plant-fed fish has been found to be adequate in short feeding studies [7], but concern about potential negative health effects from natural toxins and anti-nutritional factors including phytoestrogens remains [4,8]. Some anti-nutritional factors are considerably resistant against heat and digestion and have the potential for carry-over into the food chain. Several studies have shown that bioactive compounds may affect physiological functions in animals and humans including negative effects on intestinal health [9]; however, information for fish is limited [4]. The potential transfer of undesirable substances from new sources of aquafeeds might thus lead to potential health risks for consumers of fish products [10]. The assessment of transmissibility requires analytical methods that can be reliably applied for the detection of relevant natural contaminants in agricultural crops, and the considerable prevalence of mycotoxins and phytoestrogens makes them priority target analytes. However, only a few recent studies have surveyed mycotoxin levels in fish feed or farmed fish [11,12,13,14,15,16], and phytoestrogens are even less investigated [17,18]. 

There is a risk of mycotoxicosis in farmed fish due to the presence of mycotoxins in plant feed ingredients, but information on effects in fish is limited [11,19]. Mycotoxins comprise a large variety of secondary metabolites produced by fungi such as *Fusarium* spp., *Aspergillus* spp., *Alternaria* spp. and *Pencillium* spp. that infect agricultural crops both in the field and during storage, depending on their preferred growth conditions [20]. The presence of mycotoxins in practically all feed- and foodstuffs worldwide, although at different levels, is critical for nutritional security and safety, and important for animal and human health and welfare [21]. In moderate climate zones, major mycotoxin classes associated with *Fusarium* crop infections are trichothecenes, zearalenones and enniatins. The most important trichothecenes (polycyclic sesquiterpenoids) are A-type HT-2 toxin (HT-2) and T-2 toxin (T-2) and B-type deoxynivalenol (DON), including the acetylated and glucosidated derivatives 3-acetyl-deoxynivalneol (3-ADON), 15-acetyldeoxynivalenol (15-ADON) and deoxynivalenol-3-glucoside (DON-3G), as well as nivalenol (NIV). Furthermore, the mycoestrogen zearalenone (ZEN) shows considerable occurrence and toxicity. The ionophoric enniatins (ENN) B, B1, A, and A1 are detectable in almost all grain samples and considered an emerging threat [22]. In contrast, toxicity caused by ergot alkaloids such as ergosine, ergonovine, ergotamine, ergocristin, ergocornine and α-ergocryptine in *Claviceps purpurea*-infected cereals has been known as ergotism for centuries. Ergot contamination is a sporadic issue but appears to have increased in recent years. The storage mycotoxin of main concern in Nordic countries is ochratoxin A (OTA), a pentaketidic isocoumarin produced by *Penicillium* or *Aspergillus* sp. In contrast, aflatoxins and fumonisins normally do not occur in Norwegian feed commodities [23]. The European Commission has recommended maximum levels for important mycotoxins in different feed commodities [24]. Fish ingredients and composite fish feed are not specifically mentioned but the guidance levels for DON (5 mg/kg); ZEN (2 mg/kg) and OTA (0.25 mg/kg) also apply to aquaculture. Additionally, an indicative value for the sum of T-2 and HT-2 (250 µg/kg) in compound feed is provided by the EU Commission recommendation [25]. Comparable values have not been established for NIV, enniatins or ergot alkaloids because of the limited occurrence and toxicity data.

Phytoestrogens are plant-derived polyphenolic non-steroidal compounds with structural and functional similarity to animal oestrogens, which can bind to oestrogen receptors and activate oestrogen receptor-dependent pathways in mammals and fish [26]. Thus, they have the potential to disrupt the endocrine system by competing with endogenous hormones. Phytoestrogens can be broadly differentiated into isoflavones, coumestans and lignans, depending on the alkylation pattern in the basic isoflavone molecule structure [27]. Legumes, especially soy, are rich in isoflavones, which occur in plants mainly in glucosidated form, whereas the unconjugated molecules are prevalent after uptake. Important representatives of this substance class are the glucosides daidzin, genistin, glycitin and their respective free counterpart’s daidzein, genistein and glycitein [28]. They are also potential substrates for metabolic glucuronidation or sulphatation reactions in the liver and kidneys due to the hydroxyl groups in the molecule and could be excreted as conjugates [29]. Processed soy protein concentrates have an increased aglycon content, which results in improved phytoestrogen absorption from the diet [30]. Exposure of fish to phytoestrogens in feed has been shown to cause reproductive effects and to affect growth and metabolism [31], but the levels in the edible tissue of soy-fed fish and potential human exposure have not been investigated so far. 

The assessment of possible health risks from the consumption of fish fed with plant-derived feed requires the development of appropriate analytical methods for the detection of transferred contaminants and bioactive compounds. Mycotoxins are usually analysed by liquid chromatography tandem mass spectrometry (LC-MS/MS) with different multi-toxin methods and in various matrices such as bulk cereals, flour, nuts, food products and hay bales [32,33,34,35,36,37,38,39,40]. Advanced sampling schemes and extraction protocols have been developed, resulting in improved homogeneity and recovery so that method validation can be performed [41]. Sample preparation often includes single-step solvent extraction using acidic acetonitrile/water mixtures, followed by solid-phase extraction (SPE) or immunoaffinity purification [39]. Matrix effects can be controlled by using matrix-matched calibration and isotope-labelled internal standards (ISTD), which are available for trichothecenes but not for enniatins and ergot alkaloids [32,33,36,37,38,40]. Notably, fewer LC-MS/MS methods have been described for ergot alkaloids than for *Fusarium* toxins, focussing on rye, feed and seeds as typical matrices [34,37]. In contrast, phytoestrogens are mostly measured in physiological samples including human and animal plasma, milk and urine in connection with monitoring of dietary exposure [42,43]. The LC-MS/MS methods developed for the detection of phytoestrogens in soy and food items use methanol‒water extraction and reversed-phase (RP) chromatography [44,45]. 

Earlier studies have measured several mycotoxins in feed ingredients, aquafeeds and fish fillets [11,13,14,16,46] but ergot alkaloids were not among the analytes. In addition, we have found one report of the occurrence of phytoestrogens in foods of animal origin, including a few fish samples [47]. Considering the potential consumer health risk resulting from the extensive introduction of agricultural crops into fish feed and contaminant carry-over, analytical methods for the reliable detection of natural toxins and bioactive compounds are required. The present study was thus intended to fill this gap by developing a multiplexed LC-MS/MS method for the simultaneous quantification of 25 relevant feed-borne mycotoxins and phytoestrogens in feed and fish.

## 2. Results and Discussion

### 2.1. Fish Feed with Fixed Contents of Wheat Gluten, Soy Protein or Pea Protein

Finished feed has to comply with national and international legislation regarding maximum contents of certain contaminants including some mycotoxins [24,25]. In the present study, the fish diets were prepared in a fully equipped feed technology research facility based on materials that are commonly used in Norwegian aquaculture. Since the focus of the fish experiments was the potential transfer of natural contaminants from feed into fish, and not digestibility or feed utilisation, the composition was balanced with regard to plant-based ingredients (Table 1). Constant levels of 15% or 30% wheat gluten, soy protein concentrate or pea protein concentrate were achieved by adjusting the amount of fishmeal, which resulted in slight differences in the total crude protein and total lipid contents between the diets (Table 1). By keeping the ratio of plant-derived ingredients constant, comparability of the analytical results for the targeted metabolites was ensured.

### 2.2. Exposure of Zebrafish and Salmon to Plant-Derived Aquafeeds

The zebrafish and salmon included in the feeding experiments showed an overall normal growth performance (data not shown). Observable differences in growth rate between diet groups in on-growing salmon in the order of SPC15 > SPC30 > WG15 ≈ FM > WG30 were small but proportional to the feed intake by the same groups. The zebrafish study also included an exposure to PPC15 and PPC30 feed compositions, resulting in a slight growth reduction that had previously also been described for rainbow trout [48]. We considered, however, that the small weight gain differences observed in the present study would not significantly affect the analysis of potentially transmitted contaminants in fish muscle.

### 2.3. Characteristics of Targeted Analytes in Method

**Figure 1:**
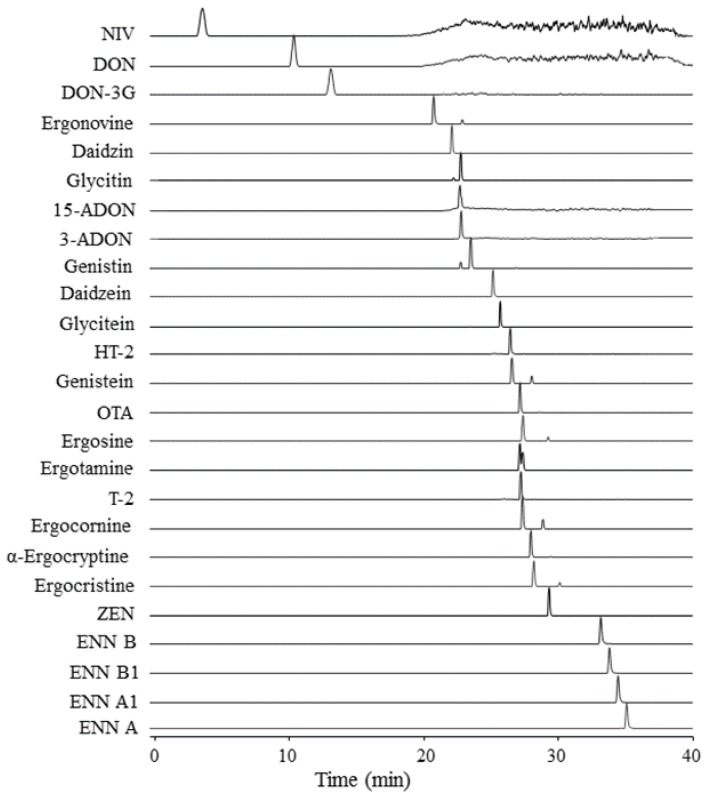
Chromatograms of targeted analysis of 100 µg/L in solvent of the 25 mycotoxins and phytoestrogens included in the multi-analyte LC-HRMS/MS method.

The mycotoxins and phytoestrogens included in the multi-analyte LC-HRMS/MS method had considerable differences in their molecular weights and structures (Table S1). Furthermore, there were sizeable differences in compound solubilities, e.g., between the hydrophilic DON, DON-3G, 3-ADON, 15-ADON and NIV and the lipophilic enniatins. These differences, as reflected by the logP (Table S1), became obvious in the order of retention on the reversed-phase LC-column (Figure 1). Molecular structure and logP were obtained from the PubChem database (https://pubchem.ncbi.nlm.nih.gov/). Retention times differed with up to 30 min under the optimised chromatographic conditions of the ammonium acetate/MeOH gradient, while peak widths were small demonstrating good signal resolution. MeOH proved to be the best eluent for combining the different analytes in one LC method. Previous studies have shown that MeOH improves peak shape and sensitivity in the analysis of trichothecenes [32,33,35,37,38,39] and the same solvent has been used for phytoestrogen chromatography [45].

During method development, all compounds were analysed in positive and negative ESI mode for the determination of the highest peak intensities and best target ions, which included proton, ammonium, sodium and acetate adducts (Table 2). The HRMS/MS parameters were adjusted accordingly so that each compound was measured in targeted analysis under optimal conditions.

### 2.4. Optimisation of Sample Preparation

**Figure 2:**
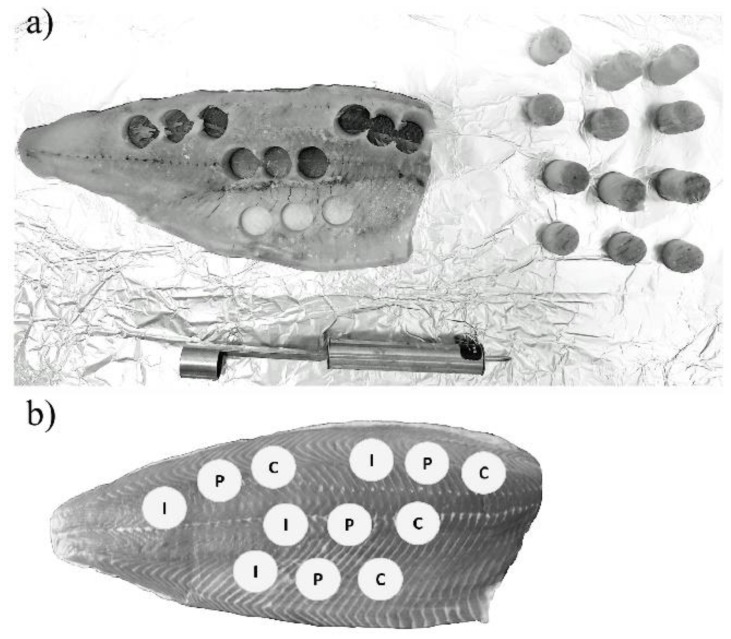
(**a**,**b**) Sampling scheme for homogenous sampling of representative aliquots from a salmon fillet. C: samples used for the chemical analyses in the present study. P and I: samples used for proteomic and immunological analyses in the same project.

Appropriate sampling and sample extraction are prerequisites for the reliability of analytical methods [39,40,41]. Several studies describing sampling strategies for the mitigation of uneven contaminant distribution in different matrices have been published [34]. Sampling plans should aim at achieving pragmatic fit-for-purpose results, providing homogeneity while limiting sample sizes and numbers. In the present experiment, potential distributional heterogeneity was not an issue in the preparation of zebrafish samples since the whole carcasses of three fish were ground and extracted together. In contrast, the salmon fillets were of considerable size and could not be processed in total. Consequently, we attempted to obtain representative samples by punching out tissue at different places in fillet and combining aliquots after grinding (Figure 2a). Additional tissue punches were gathered for proteomic and immunological analyses that were foreseen for subsequent studies (Figure 2b). The composite diets had already a high degree of homogeneity due to the production process. We assumed therefore that the targeted analytes were evenly distributed in samples taken from a few places in the storage bags and ground together.

Matrix effects impairing analytical method performance can be managed by using clean-up procedures, sample extract dilution, precipitation, filtering, matrix-assisted standard calibration curves and stable-isotope labelled ISTD [34,39]. Clean-up during sample preparation may include passing the extract through immunoaffinity columns or solid-phase extraction (SPE) cartridges, which can be filled with a variety of adsorbents. In the present study, we have not applied clean-up methods during sample preparation to avoid the potential loss of target analytes from surface adhesion. Additionally, the different molecular properties of the 25 compounds would optimally require the use of specific SPE materials. We have therefore attempted to develop a generally applicable sample preparation method by diluting the homogenised material with eight- to tenfold excess of adjusted solvent and using a one-step extraction procedure with subsequent submicron filtering. 

**Figure 3:**
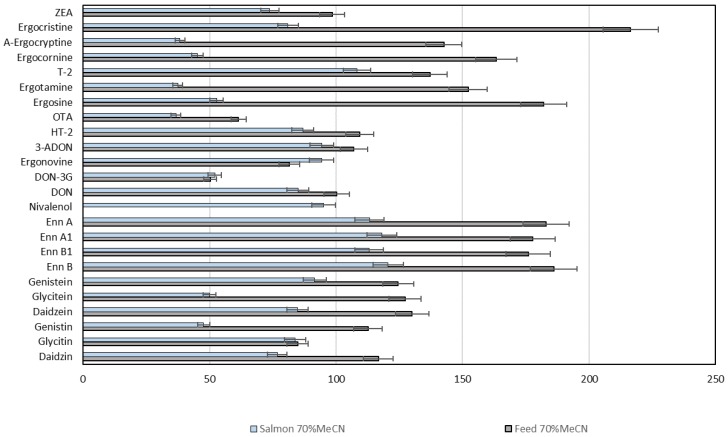
Recovery rates from spiked fish and feed matrices for the mycotoxins and phytoestrogens included in the multi-analyte LC-HRMS/MS method using optimised extraction solvent.

Extraction conditions were optimised in a number of preliminary trials by determining recovery rates from spiked matrices with different acidic MeCN/water solvent compositions and, additionally, with a two-step MeCN/water approach [36,40]. However, the two-step extraction produced multiple aqueous and organic layers in the extract, making separation difficult and decreasing analyte recovery. The overall best results for the extraction of the target analytes from feed and fish were achieved with acidic MeCN/water (70:30) (Figure 3), similar to what has been described for other multi-mycotoxin methods [36,37]. This solvent was also suitable for the phytoestrogens that have been extracted with MeOH/water in previous studies [44,45]. 

### 2.5. Performance of the Multi-Analyte LC-HRMS/MS Method

The performance characteristics of the new LC-HRMS/MS method for 25 mycotoxins and phytoestrogens were determined with regard to international standardised guidelines [49,50]. The specificity of the method for the selected analytes was excellent due to the high mass accuracy in full scan mode and targeted fragmentation (dd-MS^2^) (Figure 1; Table 2). The total run time was slightly increased in comparison to other multi-mycotoxin methods [32,33,35,37,38,39,41], leading to good chromatographic separation of the analytes. The high resolution of the analysis allowed us to resolve between isomers such as 3-ADON and 15-ADON, which previously has been sometimes a challenge [41]. 

The 25 analytes were detected with different sensitivities in fish and feed matrices differed considerably between the 25 analytes. The salmon matrix-assisted standard calibration curves showed high sensitivities for the enniatins, ZEN and the phytoestrogens daidzein and genistein, whereas the curve slopes were less steep for the trichothecenes, OTA, ergot alkaloids and remaining phytoestrogens. Interestingly, this order was not identical for solvent, zebrafish and feed matrices, comparable to results reported for other multi-mycotoxin methods that achieved different analyte sensitivities in matrices such as fruit, yoghurt, soya, hazelnut, pepper, wheat, maize, oat, rice, pasta and bread [33,35,36,37,38]. The effect of the signal enhancement or suppression by a specific matrix type can be illustrated by the connected SSE% value. Matrix impact is considered as insignificant for SSE 80‒120%, while lower values indicate significant signal decrease and higher values signal increase [32,33,35,37,38,39,40]. In the present study, SSE varied from 67% to 115% for control fish feed, 58% to 173% for salmon, and 89% to 181% for zebrafish, with ENN A showing the highest signals in the feed and fish matrices (Table 3). Considering all analytes, the feed matrix generally suppressed signals, whereas the fish matrix caused signal enhancement. 

Linearity of the standard calibration curves in different matrices was achieved for all analytes in the range 1.0 to 200 µg/L, with the exception of NIV, OTA, DON-3G and 15-ADON that were linear in the range 5.0 to 200 µg/L. The correlation coefficients (Table 2) were *R*^2^ > 0.98 for all calibration curves, irrespectively of whether or not stable-isotope labelled ISTD, similar analogue-ISTD or no ISTD were included. Considering the eight times or 10 times sample dilution during matrix extraction, the linear ranges corresponded to 8.0 (40)–1600 µg/kg for feed and salmon and 10 (50)–2000 µg/kg for zebrafish.

The limits of detection (LOD) and quantification (LOQ) in solvent, fish feed, salmon and zebrafish matrices are presented for the undiluted commodities (Table 3). The LOD ranged in solvent from 1 µg/L for ENN A1, B, B1 and genistin to 19 µg/L for NIV, in fish feed from 6 µg/kg for 15-ADON to 85 µg/kg for ENN A, in salmon from 21 µg/kg for glycitein to 144 µg/kg for NIV, and in zebrafish from 8.0 µg/kg for ergonovine and α-ergocryptine to 176 µg/kg for DON-3G. The corresponding LOQ were, as per the definition, 3.3 times higher (Table 3). The values were similar to data shown for comparable multi-mycotoxin methods. LOD ranging from 5.4 to 24 µg/kg for DON, 36 to 50 µg/kg for 15-ADON, 2.8 to 50 µg/kg for NIV, 0.2 to 47 µg/kg for ZEN, 1.0 to 18 µg/kg for T-2, and 0.7 to 12 µg/kg were reported in a number of different matrices [32,35,36,37,38]. In contrast, two methods that had been specially developed for the analysis of phytoestrogens in food products had established group LODs of, respectively, 250 µg/kg [44] and 15 µg/kg [45]. 

The precision of our multi-analyte LC-HRMS/MS method was demonstrated on the one hand by good day-to-day congruency of the solvent and matrix-assisted standard calibration curves. The coefficients of variation (% CV) for all data points in six independent experiments were generally less than 20% in solvent and less than 25% in feed, salmon and zebrafish matrices (data not shown), which was well within the guidance criteria [49]. On the other hand, precision was also assessed by intra-day and inter-day analysis of spiked quality control samples. The total within-laboratory precision was in the range of 1% for ZEN and ENN A to 17% for NIV in the feed matrix and 1% for ergonovine to 41% for NIV in the salmon matrix (Table 3). The precision data were comparable to values reported for other multi-mycotoxin methods in a variety of matrices [32,35,37,38,41]. Published precision data for phytoestrogen analysis in food commodities are scarce. When control samples were analysed using standard calibration in solvent, intra-day and inter-day% CV in the range of 1–13% were reached for a number of analytes [45].

Recovery rates in fish feed ranged from 19% to 161% for all mycotoxins and phytoestrogens in the newly developed method, with the exception of DON-3G, NIV, ergosine, ergotamine, ergocornine and α-ergocryptine that were retrieved less efficiently, and ENN A and ENN A1 that showed enhanced recoveries (Table 3). In the salmon matrix, the analytes were recovered with 69–127% except for a reduced performance for NIV and enhancement for genistein. In the zebrafish matrix, recovery rates of 41–98% were reached, except in DON-3G and NIV, which showed reduced values. The recovery rates established in the present study were similar to those determined with comparable methods ranging from 50% to 150% for a number of mycotoxins [32,35,36,37,38,39,40,41]. For phytoestrogens, recoveries between 89% and 107% in spiked solvent have been reported [45]. However, in different food matrices the rates were widely varying and in part very low, which is in strong contrast to our new LC-HRMS/MS method, showing remarkably low interference for phytoestrogen analysis in the three matrices considered (Table 3). Spiking experiments are widely used for the determination of recoveries in the validation of analytical methods, although they only can emulate naturally-contaminated samples to a certain extent. Preferably, the accuracy should be verified with a certified reference material, but this is currently not available for all target analytes and selected matrices of the LC-HRMS/MS method.

### 2.6. Mycotoxins and Phytoestrogens in Fish Feed, Zebrafish and Salmon Tissues

The in-house-validated multi-analyte LC-HRMS/MS method was used for the analysis of the customised fish feed and dietary exposed salmon and zebrafish. The feed analysis did not detect any of the targeted mycotoxins, with the exception of ENN B that was found in concentrations close to LOD in WG30 (data not shown). Norwegian aquafeeds ingredients contain generally only low amount of mycotoxins [13,23]. The highest mean contents were found in wheat (DON: 94 µg/kg; T-2+HT-2: 28 µg/kg) and maize (ZEN: 246 µg/kg), which was in compliance with the recommended maximum levels [24,25,51]. Considering that in the present study, the feed contained a maximum of 42% wheat-derived components (WG30) (Table 1), we did not expect sizable levels in the five diets. In contrast, survey data for finished feeds from Central Europe and Asia contained on average 165 µg DON/kg, 188 µg ZEN/kg and 2 µg OTA/kg [11]. Interestingly, our finding of ENN B in WG30 diets is in line with the relatively high prevalence of enniatins in cereals in Northern Europe. ENNs have shown considerable toxicity in in vitro studies and in mice [52]. Carry-over of ENN B and B1 from poultry feed into eggs has been demonstrated [22], but maximum levels for animal feed have not been established yet. 

In view of the low mycotoxin content (<LOQ) in the customised feeds in the present study, we consequently did not detect any of the targeted analytes above the respective LOQ in salmon or zebrafish tissues. There were, however, traces of ENN B in several of the WG30-exposed salmon at concentrations close to the LOD, suggesting the carry-over potential of enniatins. A relatively high occurrence of ENNs, especially ENN B, in fish muscle and livers has been previously reported [22,53] and correlates with our data. Transfer of mycotoxins such as DON, T-2 and OTA from low-level contaminated wheat gluten-containing feed into fish fillets has also been demonstrated [13]. In contrast, when salmon was fed with diets containing 2 and 6 mg DON/kg or 0.8 and 2.4 mg OTA/kg for eight weeks, up to 19 µg DON/kg was measured in the muscle, whereas up to 5 µg OTA/kg was detectable in the fish livers [46]. Human exposure following high consumption of salmon fillets with the highest DON concentrations was estimated to amount to only 2% of the established tolerable daily intake (TDI) [46,54]. Consequently, our results in the present study show that the use of plant-based fish feed containing mycotoxins below the recommended maximum levels results in negligible health risks for consumers.

The phytoestrogen analysis of the diets included in the salmon and zebrafish feeding experiments showed dose-dependent levels of all targeted analytes in the soy protein containing feeds (data not shown). Mean concentrations ranged in SPC15 from 21 µg glycitein/kg to 786 µg daidzin/kg and in SPC30 from 40 µg glycitein/kg to 1356 µg daidzin/kg. Glucosidated forms occurred in higher concentrations than the corresponding aglycons, whereas an increase of the free form had been previously observed in extruded protein preparations [30]. In PPC15 and PPC30, 26 and 54 µg glycitein/kg were detected, respectively, confirming results from a screening study on fruits and vegetables [45]. Phytoestrogen levels in food and feed are not regulated so far, and the health risks or benefits of dietary exposure in humans and animals are still under discussion [28,44]. Still, considerable oestrogenic and thyrogenic activities have been determined in vitro in commercial Spanish fish feeds [18], and further evaluation is required. A survey of the phytoestrogen content in food products of animal origin detected the highest concentrations in soy-containing milk products and farmed salmon contained up to 40 µg/kg [47].

In the present experiment, we did not find phytoestrogen concentrations above LOQ in dietary exposed zebrafish or salmon, not even in the respective SPC30 groups. Information on the uptake of isoflavones in fish is not available, but considerable differences in bioavailabilities and biotransformation are reported for warm-blooded vertebrate species [55]. We have recently studied the metabolism of isoflavones in salmon liver microsomes (article in progress) and characterised the major metabolites. Chromatographic peaks corresponding to the retention times and m/z of these metabolites were, however, absent in the muscle of the dietary exposed fish suggesting an efficient detoxification mechanism and excretion of isoflavones without accumulation in the edible parts of fish. Equol, an intestinal metabolite of daidzein, has not been studied in our experiment. Previous studies have suggested that isoflavone metabolisation by the intestinal microbiome varies considerably between producers and non-producers of equol [55]. When gibel carp (*Carassius auratus gibelio*) were exposed to 40–400 mg daidzein/kg in feed, the unchanged compound was recovered with 128 and 261 µg/kg in the fish muscle [56]. In contrast, equol was not found in any of the samples suggesting that fish could lack the necessary gut bacteria. Considering that the highest daidzein level in our experiments was with 0.2 mg/kg in SPC30, about 200-fold smaller than the lowest feed concentration in the gibel carp study, and considering the LOQ of the LC-HRMS/MS method in the fish matrix, the non-detectability of the targeted isoflavones in the salmon fillets was conclusive. However, we intend to investigate the metabolic fate of important isoflavones in fish in depth in a follow-up study.

## 3. Conclusions

The increasing use of vegetable ingredients in aquafeeds has motivated risk evaluations for mycotoxin exposure of farmed fish, which has resulted in the establishment of recommended maximum levels. Furthermore, the potential consequences of the presence of bioactive compounds such as isoflavones in plant-based feed should be monitored. We have therefore developed and validated a 25-in-1 LC-HRMS/MS method that is suitable for the survey of compliance to feed regulations and for the detection of undesirable compounds in fish fillets. The new method has excellent specificity for all analytes, while there are some differences in sensitivity due to the great diversity of molecular structures. The LOD and LOQ in fish feed, zebrafish and salmon matrices are sufficient to ensure that mycotoxin and phytoestrogen levels are below concentrations that might cause negative health effects. The accuracy of the method, described by precision and recovery of the included analytes, is satisfactory, confirming its applicability for screening and surveillance purposes. The applicability range is limited at present, however, due to the exclusion of aflatoxins. They will be added during the planned extension of the multi-analyte method. In zebrafish and salmon exposed to customised feed containing up to 30% wheat gluten, soy or pea protein concentrate, carry-over of mycotoxins or phytoestrogens could not be detected, confirming that fillets from fish fed commercial plant-based diets are safe for consumption. 

## 4. Materials and Methods 

### 4.1. Chemicals

LC-MS grade acetonitrile (MeCN), methanol (MeOH) and water (Optima, LC/MS grade,) were provided by Fisher Scientific (Loughborough, Leics., UK), and ethanol (EtOH) was obtained from VWR International (Lutterworth, Leics., UK). Acetic acid (CH_3_COOH) (>99.8%), formic acid (HCOOH) (>98%) and ammonium acetate (CH_3_COONH_4_) (>98%) were purchased from Merck KGaA (Darmstadt, Germany). 

The mycotoxins deoxynivalenol (DON), 3-actetyl-deoxynivalenol (3-ADON), nivalenol (NIV), T-2 toxin (T-2), HT-2 toxin (HT-2), zearalenone (ZEN), deoxynivalenol-3-glucoside (DON-3G), 15-acetyl-deoxynivalenol (15-ADON), ochratoxin A (OTA), ergosine, α-ergocryptine and ergocristine as well as the stable isotope-labelled analogues U-[^13^C-15]-NIV, U-[^13^C-15]-DON, U-[^13^C-21]-DON-3G, U-[^13^C-17]-3ADON, U-[^13^C-17]-15ADON, U-[^13^C-22]-HT-2, U-[^13^C-24]-T-2, U-[^13^C-20]-OTA, U-[^13^C-18]-ZEN were provided by Romer labs (Tulln, Austria) as solutions in MeCN, ranging from 10 to 100 mg/L. Intermediate standard solutions at 10 mg/L were prepared for DON-3G and 15-ADON by dilution of stock solutions with MeCN. The enniatins A, A1, B, and B1 (ENN A, A1, B, B1), ergonovine, ergotamine, ergocornine, methysergide maleate salt (MetErg) and bromocriptine mesylate (BromCri) were provided as solids by Sigma-Aldrich (St. Louis, MO, USA). Stock solutions in MeOH or MeCN were prepared for ergot alkaloids in the range of 100 to 500 mg/L, and for enniatins with 200 mg/L in MeOH. A combined intermediate standard solution with 10 mg/L was prepared for both enniatins and ergot alkaloids by combining appropriate aliquots of stock standard solutions, evaporating the mixture with a gentle stream of nitrogen and re-dissolving in MeCN/water (50:50). Finally, a combined standard solution containing all mycotoxins (Set A) was prepared by combining aliquots of stock or intermediate standard solutions, evaporating the solvent and re-dissolving in the appropriate volume MeCN/water (50:50) to obtain final concentrations of about 200 µg/L (200.0–200.12 µg/L, depending on the stock solution provided by the manufacturer). 

The phytoestrogens daidzin, genistin, glycitin, daidzein, genistein, and glycitein were bought in crystalline form from Sigma-Aldrich (St. Louis, MO, USA), and stock solutions were prepared in MeOH or DMSO (glycitein) ranging from 500 to 1000 mg/L. Individual intermediate standard solutions at a concentration of 5 mg/L were prepared by dilution with MeOH. A combined standard solution (Set B; 200 µg/L) containing all phytoestrogens was prepared by further dilution in MeCN/water (50:50). The finished Set A and Set B solutions were stable at −20 °C for several months and used for the preparation of standard calibration curves. 

Additionally, a 25-in-1 multi-analyte mixture was prepared and used in spiking experiments. All analytes were combined with regard to the concentrations of their respective stocks or intermediate standard solutions so that a final concentration of 25 µg/L per analyte was reached after spiking into feed, zebrafish and salmon samples. The multi-analyte mixture was evaporated and re-dissolved in MeCN/water (50:50). It was stable at −20 °C for about a month. 

A combined internal standard (ISTD) solution for 15 mycotoxins, containing stable isotope-labelled analogues and the ergot homologues MetErg and BromCri, was prepared in MeCN/water (50:50) to reach final concentrations of 251 µg/L U-[^13^C-18]-ZEN, 500 µg/L U-[^13^C-22]-HT-2, 443 µg/L U-[^13^C-22]-T-2, 506 µg/L U-[^13^C-15]-DON, 502 µg/L U-[^13^C-17]-3ADON, 500 µg/L U-[^13^C-17]-15ADON, 500 µg/L U-[^13^C-20]-OTA, 530 µg/L U-[^13^C-15]-NIV, 530 µg/L U-[^13^C-21]-DON-3G, 624 µg/L BromCri and 500 µg/L MetErg. The different concentrations were chosen with regard to the respective measurement sensitivities in the developed multi-analyte LC-HRMS/MS method. The ISTD solution was stored at −20 °C, adjusted to room temperature (RT) and mixed thoroughly prior to use. It was added in a ratio of 1:5 to the study samples. 

### 4.2. Preparation of Fish Diets

Diets with definite amounts of wheat gluten, soy protein concentrate or pea protein concentrate were produced at Nofima Feed Technology Centre, Fyllingsdalen, Norway. The diets were based on fishmeal (FM) as main protein source, which was replaced by 15% or 30% plant proteins. All diets contained 12% wheat that was required for binding in the extrusion process, in addition to minor inclusion of wheat as carrier for some of the additives used (Table 1). In total seven diets were produced: (1) control feed (FM), (2) 15% soy protein concentrate (SPC15), (3) 30% soy protein concentrate (SPC30), (4) 15% wheat gluten (WG15), (5) 30% wheat gluten (WG30), (6) 15% pea protein concentrate (PPC15), and (7) 30% pea protein concentrate (PPC30). The ingredients used for the preparation of diets included FM Norsildmel AS (Bergen, Norway), SPC from Agilia A/S (Videbæk, Denmark), PPC from AM Nutrition AS (Stavanger, Norway) and WG from Tereos Syral (Marckolsheim, France). All diets had an inclusion of 4% fish oil at extrusion. The feed were produced on a pilot scale twin-screw, co-rotating Wenger TX 52 extruder (Wenger, Sabetha, KS., USA) with a die of 2.5 mm diameter. After extrusion, the diets were dried for 40–70 min in a carousel dryer (Paul Klöckner, Verfahrenstechnik GmbH, Hachenburg, Germany) at 65 °C to a water content of 7–8%. The salmon diets 1 to 5 were, in addition, oil-coated with 16% fish oil after extrusion by vacuum-coating (Dinnissen, Sevenum, Netherlands) to meet the standard dietary inclusion of oil for the fish size studied. The salmon feed had a pellet size of 3.5 mm, while the zebrafish feed were ground and sieved to a pellet size of 0.6–0.8 mm.

### 4.3. Feeding Studies in Zebrafish and On-Growing Salmon

#### 4.3.1. Zebrafish

Four-month-old zebrafish (*Danio rerio*) (AB strain) with a mean weight of 0.214 g were distributed into 28 tanks (*n* = 16) and were maintained in a flow-through system with 20 % water exchange per hour (ZebTEC Stand-Alone Toxicology Rack, Techniplast, London, UK) under daily-monitored standard husbandry conditions, including a stable temperature of 28 ± 0.5 °C, pH 7.5, water conductivity of 1500 µS/cm and photoperiod of 12 h light:12 h dark at the Faculty of Biosciences and Aquaculture, Nord University, Bodø, Norway. The feeding study included 336 fish that were distributed into the system’s 3.5-litre tanks according to the seven experimental diets. Four replicate groups per diet, each consisting of 12 fish (six per gender) in one tank (and an additional four fish to compensate for potential losses during the study period), were hand-fed twice daily with a total feed amount equal to 2.5% of their body weight over a period of 46 days. The feeding behaviour and health and welfare of the fish were regularly controlled. At the end of the study, the fish were not fed for 24 h prior to sampling. They were separated by gender and euthanised individually by transfer into a tank containing a lethal dose of 200 mg/L tricaine methanesulfonate (MS222) (Sigma-Aldrich, St. Louis, MO, USA), buffered with an equal amount of sodium bicarbonate. The liver, spleen and intestines were carefully dissected under a light microscope and immediately frozen in liquid nitrogen along with the rest of the carcass. All samples were stored at −80 °C for further analyses.

The zebrafish feeding study was conducted in compliance with the guidelines provided by the Norwegian Animal Research Authority (FOTS ID 12581, 27 July 2017) and approved by the Nord University (Norway) ethics committee.

#### 4.3.2. Salmon

One-year-old post-smolt Atlantic salmon (*Salmo salar*; salmo breed strain) with a mean weight of 223 g were randomly distributed into 15 experimental tanks (1 m^3^; *n* = 32) filled with seawater at the Nofima Research Station, Sunndalsøra, Norway. The oil-coated diets 1–5 were given to randomised triplicate tanks by automatic disc feeders. Excess feed was collected once daily for calculation of feed intake. The water temperature was maintained at an average of 10.6 (±0.6) °C. The oxygen level at the tank outlets was higher than 90% at study start and about 80% at the study’s end. The water flow in each tank was set to 20 L/min.

The feeding was conducted for nine weeks. At the start of the experiment, 15 fish were sampled, and the muscle, liver and intestine were collected. After five weeks, muscle was sampled from one fish from each tank of the FM, SPC30 and WG30 groups. At the termination of the study, five fish from each tank were collected and weighed. The sampled fish were anaesthetised with 60–80 mg/L MS222, transferred and euthanised with a double dose (120–160 mg/L) MS222. Blood was drawn from the caudal vein using 2.5-mL vacutainers (VACUETTE^®^ 2.5 mL Z serum separator clot activator; Greiner Bio-One, Kremsmünster, Austria) and centrifuged at 2500× *g* for 15 min at 4 °C (Allegra 6R Centrifuge, Beckman, Indianapolis, IN, USA), and sera were stored at −20 °C. The livers and intestines of the fish were removed, and tissue samples were frozen with liquid nitrogen and stored at −80 °C. Fillets were stored at −20 °C. The remaining fish in each tank were weighed in bulk, and their mean weight was calculated, including the sampled fish. 

The salmon feeding study was performed in compliance with the national regulations for the use of animals in experiments [57]. The experiment was classified as not requiring a specific license [58] as none of the planned experimental treatments were expected to cause any distress or discomfort for the fish.

### 4.4. Extraction of Fish Feed, Zebrafish and Salmon Samples

#### 4.4.1. Fish Feed

Fish feed pellets were homogenised with a grinding mill (Retsch, Haan, Germany), and 2.5 g were weighed into 50-mL polypropylene tubes. After the addition of 20 mL extraction solvent, the samples were vortexed for 1 min, extracted on a horizontal shaker (Edmund Bühler, Tübingen, Germany) with 200 min^−1^ at room temperature (RT) for 30 min, and centrifuged with 2000× *g* for 10 min at 4 °C (Beckman Coulter, Brea, CT, USA). The supernatants were transferred into fresh 50-mL tubes and let to settle overnight (ON) at 4 °C. Subsequently, 0.5 mL of the supernatants were centrifuged for 1 min at 20,000× *g* through 0.22 µm nylon filters (Costar Spin-X; Corning, Inc., Corning, NY, USA) and 40 µL of the filtrates were transferred into LCMS vials. Finally, 10 µL ISTD solution were added to each vial. Samples were store refrigerated until analysis by LC-HRMS/MS.

The composition of the extraction solvent was optimised during method development in spiking experiments. Multi-analyte mixture (50 µL) was added to 2.5 g ground feed, which was then kept under a laminar hood for 30 min, allowing the solvent to evaporate. Extractions were performed either in one step with 20 mL acidic (0.1% formic acid (FA)) MeCN/water mixtures of different compositions (50:50; 60:40; 70:30; or 80:20) or in two steps with acidic MeCN/water (I: 80:20; II: 20:80). Based on the best recovery rates for mycotoxins and phytoestrogens, MeCN/water (70:30; 0.1% FA) was selected for all further experiments.

#### 4.4.2. Zebrafish

Three frozen, gutted zebrafish, for each replicate and diet, were thawed and, after separation of the heads, ground to a fine powder with pestle and mortar in liquid nitrogen. The powdered tissue (0.1 g) was weighed and extracted with 1 mL extraction solvent (MeCN/water 70:30; 0.1% FA). The mixture was homogenised by ultra-sonication (Branson, Danbury, CT, USA) for 10 min at 30 °C, centrifuged at 4000× *g* for 10 min at 4 °C (Thermo Scientific, Waltham, MA, USA), and the supernatant was transferred into fresh 5-mL tubes. An aliquot (0.5 mL) was filtered as described before, and 40 µL of the filtrates were transferred into LCMS vials, mixed with 10 µL of the ISTD solution, and analysed by LC-HRMS/MS. 

The recoveries of mycotoxins and phytoestrogens from the zebrafish matrix was investigated during method development by different acidic MeCN/water extraction solvents in spiking experiments with multi-analyte mixture.

#### 4.4.3. Salmon

The salmon fillets were half-thawed. Tissue pieces of equal size were sampled from four different areas using a steel puncher (0.5 cm in diameter) (Figure 2). The tissue samples were ground with a pestle and mortar, combined, and 2.5 g were transferred into a 50-mL tube, extracted with 20 mL extraction solvent (MeCN/water 70:30; 0.1% FA) and thoroughly homogenised for 40 s by ultra-turrax (Janke & Kunkel, IKA-Werke, Staufen, Germany). To avoid cross-contamination, the ultra-turrax was washed with water for 20 s between samples from the same fish tank and with water and MeOH for 40 s between samples from different tanks. The samples were vortexed for 30 s and extracted using a horizontal shaker (Edmund Bühler) with 200 min^−1^ at RT for 1 h. Subsequently, they were centrifuged with 2000× *g* for 10 min at 4 °C (Beckman Coulter), and the supernatants were transferred into fresh 50-mL tubes and let to settle overnight at 4 °C. Subsequently, 0.5-mL aliquots were filtered as described before, and 40 µL of the filtrates were transferred into LCMS vials, mixed with 10 µL of the ISTD solution, and analysed by LC-HRMS/MS. The recovery of mycotoxins and phytoestrogens from the salmon matrix was investigated as described for zebrafish.

### 4.5. Preparation of Matrix-Assisted Standard Calibration Curves

Calibration curves in solvent were prepared by evaporating 200 µL Set A solution with nitrogen and re-dissolving with 200 µL Set B, resulting in a standard solution with 200 µg/L for all 25 analytes included in this study. The standard solution was serially diluted with MeCN/water (50:50) to produce calibrants with 200, 100, 50, 10, 5 and 1 µg/L. For the preparation of the matrix-assisted standard calibration curves, 40 µL aliquots of the calibrants were transferred into LCMS vials and 10 µL ISTD solution was added. They were evaporated with nitrogen at 40 °C and re-dissolved in the same volume of blank matrix extract that had been prepared either from control feed or from zebrafish or salmon in the respective FM-control groups by pooling equal volumes of replicates. The calibration standards were transferred into LCMS vials and analysed by LC-HRMS/MS.

### 4.6. Development of the Multi-Analyte Liquid Chromatography High-Resolution Mass Spectrometry (LC-HRMS/MS) Method

Multi-analyte analysis was performed on a Q-Exactive™ Hybrid Quadrupole-Orbitrap HRMS/MS equipped with a heated electrospray ion source (HESI-II) and coupled to a Vanquish UHPLC system (Thermo Scientific). The instrument setup was similar to that described in a previous study [36]; however, there were several modifications and different analytes were included. The HESI-II interface was operated at 300 °C, alternatively in positive and negative mode during one run. The parameters were adjusted as follows: spray voltage 3.2 and 2.5 kV (positive and negative mode, respectively), capillary temperature 280 °C, sheath gas flow rate 35 L/min, auxiliary gas flow rate 10 L/min, and S-lens RF level 55. 

The Q-Exactive HRMS/MS was operated in full scan (FS) mode with the inclusion of targeted fragmentation (data-dependent MS/MS: dd-MS^2^). For full scans, the mass ranges were set to *m*/*z* 90–900 and 200–900 in negative and positive mode, respectively. FS data were acquired at a mass resolution of 70,000 full width half-maximum (FWHM) at *m*/*z* 200, while mass resolution was set to 17,500 FWHM at *m*/*z* 200 during dd-MS2. The automated gain control (AGC) target was set to 5 × 10^5^ ions for a maximum injection time (IT) of 250 ms in the FS mode, whereas for dd-MS^2^ mode the AGC target was 1 × 10^5^ and the IT was 100 ms. The inclusion list for the targeted analysis contained the *m*/*z*, retention times (RT), and normalised collision energies (NCE) (Table 2). NCE values were determined by direct infusion of standard solutions in the mobile phase (MeCN/water (50:50), containing 5 mM ammonium acetate and 0.1% acetic acid) by using a syringe pump at a flow rate of 5 μL/min. The quadrupole mass filter was operated with an isolation window of *m*/*z* 3. External mass calibration of the Q-Exactive HRMS/MS was performed every three days over the mass range *m*/*z* 90–2000, in accordance with the manufacturer’s instructions. The identification of the 25 mycotoxins and phytoestrogens included in the multi-analyte method was supported by the determination of specific retention times, fragmentation patterns and accurate masses, which were obtained using a mass accuracy window of ±5 ppm with respect to the theoretical accurate masses (Table S1). Chromatographic separation was achieved at 30 °C on a 150 × 2.1 mm Kinetex reversed-phase F5 column (2.6 µm, 100Å; Phenomenex, Torrance, CA, USA) with a 0.5 μm × 0.004” ID, HPLC KrudKatcher Ultra Column In-Line filter. The flow rate of the mobile phase was 0.25 mL/min, and the injection volume was 1 μL. Eluent A was water and eluent B was MeOH (both containing 5 mM ammonium acetate and 0.1% acetic acid). Since the solubility of ammonium acetate in MeOH is limited, it was first dissolved in 25 mL water before MeOH was added. The total run time was 43 min, and gradient elution was employed starting at 3% B for 1 min, linearly increasing to 15% B in 15 min, to 79% B in 10 min, and finally, to 100% B in 13 min. After washing the column for 2 min with 100% B, the mobile phase was returned to the initial conditions and the column was eluted isocratically for 2.5 min. The column was regularly washed with 70% methanol to prevent cross-contamination. Calibration standards and samples were analysed in randomised order and intercepted with blank solvent samples to minimise analytical bias from sample positions and to reduce sample-to-sample carry-over.

### 4.7. Validation of the Multi-Analyte LC-HRMS/MS Method

The method was validated with regard to the guidelines established by the International Organization for Standardization [49,50]. The analytical selectivity was determined by the combination of LC retention time and high-resolution mass detection including dd-MS^2^ product ion qualifying of the different analytes. Measured peak areas were used for quantification. Sensitivity for the different analytes was expressed, by the slope of the respective six-point standard calibration curves (mean of three to four independent experiments) that were calculated by linear regression analysis in both solvent (MeCN 50:50) and the different matrices. The linear range was defined as the concentration interval, in which the regression coefficient *R*^2^ was ≥ 0.96. Although internal standard calibrations were used for 15 of the analytes for the compensation of matrix interferences, potential suppression and enhancement (SSE%) of signals from the co-eluting matrix were estimated for all analytes as the ratio of the slope of the matrix-assisted standard calibration curve to the calibration curve in MeCN/water (50:50). If SSE values were above or below 100%, signal enhancement or suppression by the matrix could be assumed.

Considering the negligible noise in the extracted high-resolution mass chromatograms, the limits of detection (LOD) and limits of quantification (LOQ) of the 25 analytes were calculated based on the standard deviation of the y-intercept of the respective calibration curves and their corresponding slopes (m) as $LOD=3\times \frac{SD}{m}$, $LOQ=10\times \frac{SD}{m}$ [59]. The accuracy of the method was assessed by determining recovery by spiking experiments and precision in terms of total within laboratory precision (RSi_R_) considering intra- and interday variabilities together [60]. Furthermore, coefficients of variation (% CV) were determined for all concentration points in the solvent and matrix-assisted standard calibration curves. Recovery rates were calculated for all analytes as the mean of three to four experiments at a spiking level of 25 µg/L. In a few cases, where the matrix-assisted standard curves in feed or fish matrices did not pass through the origin but showed a positive signal on the ordinate due to background noise, this was corrected by virtually moving the curve with parallel shift on the abscissa. The corresponding concentration difference was added to the spike concentration used in the recovery experiments according to Recovery_(spike corrected)_ = (measured concentration − blank)/(spiked concentration + concentration difference to origin).

Measured results for fish feed and fish study samples were converted from concentrations (µg/L) into content in the respective matrix (µg/kg) by using the factors 0.1 for zebrafish and 0.125 for salmon and feed. 

### 4.8. Data Analysis

The Q-Exactive was calibrated using Xcalibur software, version 2.2 (Thermo Scientific). The molecular formulas and exact masses of the target analytes were calculated using the built-in Qualbrowser of the Xcalibur 2.2 software, which was also applied for signal quantification. Microsoft Excel (Version 2016, Microsoft Corporation, Redmond, WA, USA) was used for basic statistics (e.g., calculation of mean, minimum and maximum values, regression and relative standard deviation).

## Figures and Tables

**Table 1:** Composition of customised salmon and zebrafish feed (FM, fish meal; SPC, soy protein concentrate; PPC, pea protein concentrate).

Diet Composition (g/100 g)	FM (Control)	SPC15	SPC30	WG15	WG30	PPC15	PPC30
**Salmon**							
Fish meal	63.35	48.35	33.35	48.35	33.35	-	-
Wheat	12.0	12.0	12.0	12.0	12.0	-	-
Soy prot. conc.	-	15.0	30.0	-	-	-	-
Wheat gluten	-	-	-	15.0	30.0	-	-
Fish oil	20.0	20.0	20.0	20.0	20.0	-	-
Additives ^#^	4.65	4.65	4.65	4.65	4.65	-	-
*Total protein*	45.2	44.6	44.0	46.7	48.1	-	-
*Total lipids*	26.5	25.1	23.8	25.4	24.3	-	-
**Zebrafish**							
Fish meal	79.35	64.35	49.35	64.35	49.35	64.35	49.35
Wheat	12.0	12.0	12.0	12.0	12.0	12.0	12.0
Soy prot. conc.	-	15.0	30.0	-	-	-	-
Wheat gluten	-	-	-	15.0	30.0	-	-
Pea prot. conc.	-	-	-	-	-	15.0	30.0
Fish oil	4.0	4.0	4.0	4.0	4.0	4.0	4.0
Additives ^#^	4.65	4.65	4.65	4.65	4.65	4.65	4.65
*Total crude protein*	56.2	55.6	55.0	57.7	59.1	53.1	49.9
*Total lipids*	12.0	10.7	9.4	10.9	9.8	11.3	10.7

^#^ Additives: Vitamin mix (2%), Mineral mix (0.59%), Monosodiumphosphate-24% P (2%), Yttrium oxide (0.01%), Carophyll Pink-10% (0.05%).

**Table 2:** Optimised LC-MS/MS conditions and calibration curve performances (*R*^2^) for target compounds in different matrices.

Compound	Ionisation Mode	Target Ion	RT (min)	Precursor (*m*/*z*)	NCE (ev)	Fish Feed (*R*^2^)	Salmon (*R*^2^)	Zebrafish (*R*^2^)	ISTD
DON	ESI neg	[M+CH_3_COO]^−^	12.3	355.1387	17	0.9996	0.9964	0.9996	^13^C-DON
3-ADON	ESI neg	[M+CH_3_COO]^−^	23.8	397.1493	15	0.9998	0.9975	0.9999	^13^C-3-ADON
15-ADON	ESI pos	[M+Na]^+^	23.7	361.1258	15	0.9999	0.9986	0.9969	^13^C-15-ADON
DON-3G	ESI neg	[M+CH_3_COO]^−^	15.1	517.1916	17	0.9993	0.9935	0.9851	^13^C-DON-3G
NIV	ESI neg	[M+CH_3_COO]^−^	5.30	371.1337	17	0.9983	0.9901	0.9972	^13^C-NIV
T-2	ESI pos	[M+NH_4_]^+^	28.0	484.2541	15	0.9995	0.9978	0.9995	^13^C-T-2
HT-2	ESI neg	[M+CH_3_COO]^−^	26.4	483.2225	15	0.9998	0.9961	0.9998	^13^C-HT-2
OTA	ESI neg	[M−H]^−^	27.3	402.0739	32	0.9992	0.9984	0.9998	^13^C-OTA
ZEN	ESI neg	[M−H]^−^	29.5	317.1384	50	0.9999	0.9985	0.9998	^13^C-ZEN
Ergonovine	ESI pos	[M+H]^+^	21.9	326.1863	50	0.9996	0.9992	0.9999	MetErg
Ergosine	ESI pos	[M+H]^+^	27.6	548.2868	27	0.9990	0.9979	0.9999	BromCri
Ergotamine	ESI pos	[M+H]^+^	28.0	582.2711	32	0.9973	0.9985	0.9999	BromCri
Ergocornine	ESI pos	[M+H]^+^	28.1	562.3024	25	0.9992	0.9973	0.9998	BromCri
α-Ergocryptine	ESI pos	[M+H]^+^	28.8	576.3180	25	0.9993	0.9980	0.9999	BromCri
Ergoscristine	ESI pos	[M+H]^+^	29.0	610.3024	27	0.9984	0.9980	0.9999	BromCri
ENN A	ESI pos	[M+NH_4_]^+^	35.1	699.4903	27	0.9943	0.9981	0.9992	-
ENN A1	ESI pos	[M+NH_4_]^+^	34.4	685.4746	27	0.9984	0.9987	0.9991	-
ENN B	ESI pos	[M+NH_4_]^+^	33.1	657.4433	27	0.9986	0.9952	0.9998	-
ENN B1	ESI pos	[M+NH_4_]^+^	33.8	671.4590	27	0.9993	0.9987	0.9993	-
Daidzein	ESI neg	[M−H]^−^	26.1	253.0506	75	0.9993	0.9980	0.9982	-
Daidzin	ESI neg	[M+CH_3_COO]^−^	23.1	475.1246	10	0.9997	0.9984	0.9998	-
Genistein	ESI neg	[M−H]^−^	27.3	269.0455	70	0.9997	0.9986	0.9979	-
Genistin	ESI neg	[M+CH_3_COO]^−^	24.4	491.1195	10	0.9994	0.9974	0.9999	-
Glycitein	ESI neg	[M−H]^−^	26.4	283.0612	35	0.9998	0.9997	0.9989	-
Glycitin	ESI neg	[M+CH_3_COO]^−^	23.6	505.1351	10	0.9994	0.9979	0.9994	-

**Table 3:** Performance validation parameters for the multi-analyte LC-HRMS/MS method (*n* = number of analysis for each category; a: solvent; b: fish feed; c: salmon; d: zebrafish).

Compound	*n*	LOD					LOQ				SSE (%)	Total within Laboratory Precision (%)		Recovery ± SD (%)		
		(µg/L)	(µg/kg)				(µg/L)	(µg/kg)								
	(a/b/c/d)	(a)	(b)	(c)	(d)		(a)	(b)	(c)	(d)	(b/c/d)	(b)	(c)	(b)	(c)	(d)
**with ISTD**																
DON	4/4/3/3	3	23	67	22		9	78	225	74	77/87/133	4	4	90 ± 7	107 ± 13	92 ± 25
3-ADON	4/4/3/3	4	17	57	9		15	56	189	29	98/116/144	3	3	112 ± 17	96 ± 12	78 ± 20
15-ADON	4/3/3/3	5	6	43	63		16	20	142	210	96/161/141	10	3	133 ± 2	107 ± 25	86 ± 14
DON-3G	4/4/3/3	5	36	115	176		18	121	383	588	85/95/119	11	22	19 ± 9	83 ± 20	48 ± 31
NIV	4/4/3/3	19	59	144	76		63	196	479	252	71/65/115	17	41	57 ± 34	69 ± 33	41 ± 24
T-2	4/4/3/3	4	26	53	26		12	88	176	86	97/136/151	3	3	96 ± 17	99 ± 15	90 ± 19
HT-2	4/4/3/3	2	22	70	15		8	73	235	52	89/129/149	2	3	94 ± 18	96 ± 11	98 ± 15
OTA	4/4/3/3	5	41	44	21		18	138	148	68	105/139/150	6	4	75 ± 13	87 ± 23	83 ± 20
ZEN	4/4/3/3	6	11	43	14		22	38	143	47	90/125/125	1	2	109 ± 5	106 ± 18	96 ± 25
Ergonovine	4/4/3/3	6	23	35	8		19	77	115	26	85/130/106	2	1	84 ± 8	98 ± 13	87 ± 30
Ergosine	4/4/3/3	4	35	52	9		12	117	173	32	79/129/138	7	11	69 ± 27	89 ± 31	72 ± 20
Ergotamine	4/4/3/3	2	59	56	10		8	195	188	35	81/134/155	10	9	64 ± 10	84 ± 26	77 ± 18
Ergocornine	4/4/3/3	3	32	59	16		11	108	196	53	93/129/136	11	11	59 ± 16	90 ± 26	70 ± 14
α-Ergocryptine	4/4/3/3	4	15	38	8		14	50	126	28	67/119/137	7	8	53 ± 7	82 ± 21	70 ± 14
Ergoscristine	4/4/3/3	3	30	51	10		10	100	170	32	70/117/135	8	5	77 ± 22	88 ± 24	54 ± 26
**without ISTD**																
ENN A	4/4/3/3	4	85	49	32		13	284	165	108	115/173/181	1	8	161 ± 14	117 ± 27	81 ± 17
ENN A1	4/4/3/3	1	45	40	33		5	150	133	111	102/122/148	3	11	147 ± 21	110 ± 29	80 ± 16
ENN B	4/4/3/3	1	41	78	17		4	138	260	57	95/132/152	2	10	117 ± 16	107 ± 30	79 ± 17
ENN B1	4/4/3/3	1	29	40	29		5	96	133	96	102/125/147	3	12	134 ± 9	106 ± 35	79 ± 17
Daidzein	4/4/3/3	13	30	50	48		42	100	168	159	86/120/101	4	13	123 ± 9	122 ± 18	93 ± 15
Daidzin	4/4/3/3	3	19	45	15		12	62	152	51	86/113/140	4	16	93 ± 21	93 ± 6	71 ± 13
Genistein	4/4/3/3	11	20	42	52		37	66	141	172	81/120/104	2	13	114 ± 23	127 ± 28	91 ± 18
Genistin	4/4/3/3	1	29	72	11		5	95	241	35	101/143/149	4	12	101 ± 45	88 ± 1	69 ± 14
Glycitein	4/4/3/3	11	18	21	37		36	58	68	124	80/58/89	2	16	127 ± 3	118 ± 24	96 ± 16
Glycitin	4/4/3/3	4	27	51	26		13	90	170	88	94/123/121	4	23	96 ± 14	113 ± 13	97 ± 22

## Supplementary Materials

The following are available online at https://www.mdpi.com/2072-6651/11/4/222/s1, Table S1: Molecular characteristics of target analytes.
