# Supplementary material for: Development and Validation of a Liquid Chromatography High-Resolution Mass Spectrometry Method for the Simultaneous Determination of Mycotoxins and Phytoestrogens in Plant-Based Fish Feed and Exposed Fish
Source: Toxins (Basel). 2019 Apr 13;11(4):222. doi: 10.3390/toxins11040222 (PMC6520669; doi:10.3390/toxins11040222)
Supplement: Supplementary file 1 [file toxins-11-00222-s001.pdf]

Supplementary Materials: Development and Validation of a Liquid Chromatography High-Resolution Mass Spectrometry Method for the Simultaneous Determination of Mycotoxins and Phytoestrogens in Plant-Based Fish Feed and Exposed Fish

Amritha Johny *, Christiane Kruse Fæste, André S. Bogevik, Gerd Marit Berge, Jorge M.O. Fernandes and Lada Ivanova

Table S1. Molecular characteristics of target analytes.

Compound	Structure	Formula	MW (g/mol)	LogP
DON	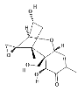	$C_{15}H_{20}O_6$	296.319	–0.7
3-ADON	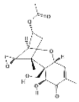	$C_{17}H_{22}O_7$	338.356	–0.1
15-ADON	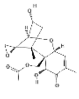	$C_{17}H_{22}O_7$	338.356	–0.7
DON-3G	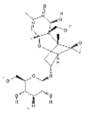	$C_{21}H_{30}O_{11}$	458.46	–2.3
NIV	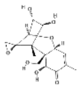	$C_{15}H_{20}O_7$	312.318	–1.7
T-2	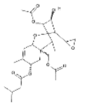	$C_{24}H_{34}O_9$	466.527	0.9
HT-2	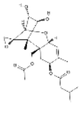	$C_{22}H_{32}O_8$	424.490	0.4
OTA	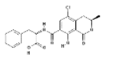	$C_{20}H_{18}ClNO_6$	403.815	4.7
ZEN	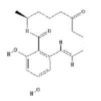	$C_{18}H_{22}O_5$	318.369	3.6
Ergonovine	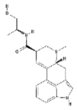	$C_{19}H_{23}N_3O_2$	325.412	1.8
Ergosine	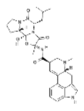	$C_{30}H_{37}N_5O_5$	547.656	1.8

Ergotamine	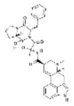	$C_{33}H_{35}N_5O_5$	581.673	2.0
Ergocornine	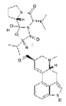	$C_{31}H_{39}N_5O_5$	561.683	2.4
α-Ergocryptine	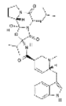	$C_{32}H_{41}N_5O_5$	575.71	2.7
Ergoscristine	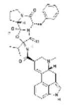	$C_{35}H_{39}N_5O_5$	609.727	3.0
ENN A	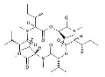	$C_{36}H_{63}N_3O_9$	681.912	7.6
ENN A1	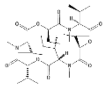	$C_{35}H_{61}N_3O_9$	667.885	7.3
ENN B	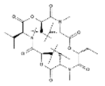	$C_{33}H_{57}N_3O_9$	639.831	6.5
ENN B1	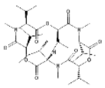	$C_{34}H_{59}N_3O_9$	653.858	6.9
Daidzein	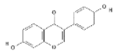	$C_{15}H_{10}O_4$	254.241	2.5
Daidzin	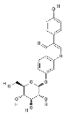	$C_{21}H_{20}O_9$	416.382	0.7
Genistein	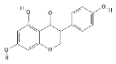	$C_{15}H_{10}O_5$	270.240	2.7
Genistin	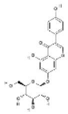	$C_{21}H_{20}O_{10}$	432.381	0.9
Glycitein	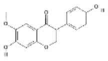	$C_{16}H_{12}O_5$	284.267	2.4
Glycitin	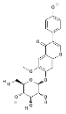	$C_{22}H_{22}O_{10}$	446.408	0.6
